# An Inaugural Thesis on Hygiene, for the Degree of Doctor of Medicine, in Atlanta Medical College

**Published:** 1860-10

**Authors:** Thomas H. Sanders

**Affiliations:** Anderson, S. C.


### ARTICLE II.

*An Inaugural Thesis on Hygiene, for the Degree of Doctor of Medicine, in Atlanta Medical College.* By THOMAS H. SANDERS, of Anderson, S. C., 1860.

There is, perhaps, no subject, however intimately connected with the science of medicine, fraught with more interest to the medical profession and public generally, than the one which we have now under consideration. And there is certainly none which offers greater inducements for investigation; for it is one which, if correctly understood and regarded, would enable us to prevent many diseases, and render life pleasant and happy. Health is what we all desire—it is what we seek, and it is that which makes life desirable; for without it, even a king upon his throne, surrounded with all his regal splendor, would be miserable, and life to him a burden. This is, therefore, a subject of common interest to every living soul. But more particularly does it interest the physician, whose duty it is to study the nature and causes of disease. Unfortunately, however, this subject has not received that attention from the members of the medical profession, which we think its importance demands. It has for its object the accomplishment of much good, and should, therefore, receive an earnest consideration. But many who bear the name of physician consider it their peculiar province to treat diseases, and have not philanthropy enough to study, and communicate the means for their prevention.—Such men mistake the object of their mission, and degrade, rather than elevate, the standard of medical science.

The daily duties of a practitioner of medicine, bring to his notice many lamentable cases wherein a knowledge of the laws of health would have prevented much suffering, and perhaps preserved life itself. Physicians therefore, who are actuated by proper motives, could do much towards the relief of suffering humanity, without the administration of one grain of medicine. But I do not of course wish to be understood as seeking to detract one iota from the science of medicine; for I believe it to be a true science, based upon correct principles, and, however humble may have been its origin, it has now risen to a position which demands respect, and claims the admiration of the world. But, unfortunately for the advancement of this noble science, there are men enlisted in her cause who have not the faintest conception of what constitutes the true physician. In the language of our eminent Professor of the Practice of Medicine: "A man should regard himself as the guardian of the public health, and not simply as an agent to administer physic. And he who so far degrades himself as to make pecuniary gain a matter of paramount consideration in the practice of medicine, is unworthy of the profession in which he is engaged."

In obtaining a knowledge of the laws of health, and making a proper use of that knowledge, we add greatly to our earthly enjoyment, and are led to a conception of the beneficence of Providence so clear as to beget within us profound gratitude for the well ordered arrangement of nature. But we are human, and are liable to fall short of what our own interest requires. We may have the means in our power to render ourselves pleasant and happy, and then often neglect to employ those means for the promotion of our comfort and happiness. And it is not until our bodies are racked with pain and disease, that we can form a true estimate of the value of health.

It is in the nursery that Hygiene plays so important a part in the prevention of diseases, and it is here that the foundation of future health may be laid, by judicious management upon the part of those who have the care and management of young children. Those who assume these responsibilities, should endeavor to acquaint themselves as far as possible, with the best means for the development of a child's physical and mental faculties. The acquisition of this knowledge would save parents many an anxious hour, and would enable them often to prevent the disorders of their children.—We trust that the diffusion of medical knowledge will enlighten the public mind upon this subject, and be the means of arresting the ravages of many diseases which destroy thousands annually. Already has the genial rays of hygiene penetrated the dark alleys of our commercial cities, and driven before it many of those pestilential diseases which affect so seriously their inhabitants—spreading joy and peace where once gloom and misery took up their abode.

The importance of attending scrupulously to the regulation of a child's diet, cannot be too strongly impressed upon the minds of mothers and nurses, as many cases of Cholera Infantum, and other disorders of children, can often be traced to some dietetic error. The milk of the mother is, of itself, sufficient to support and nourish a child until it has arrived at an age when more solid food can be properly digested. The analysis of milk has proven it to contain all the elements necessary to build up the various tissues of the body. Showing, conclusively, that it is the pabulum intended by nature for the support of the infant, until its digestive and assimilative organs had acquired sufficient vigor to appropriate more solid food to the sustenance and nourishment of the body. Nothing can be more prejudicial to the health of young children, than the custom of feeding them upon any and every kind of food; many of these, perhaps, being articles of difficult digestion, which will of necessity prove a source of derangement to the stomach and bowels, and which, if not arrested, will often terminate in serious consequences to the child.

The nervous system of young children is so susceptible to impressions made upon it, that an irritation of the alimentary canal is apt to assume an inflammatory action, and the brain, sympathizing, becomes secondarily involved. So it can be readily understood how the presence of indigestible articles of food in the stomach of a young child, may, by producing an irritable condition of this organ, become the primary cause of functional derangement of the brain. The process of dentition with children is often attended by much suffering; indeed, this may be truly called "the critical period in the life of a child." But, by judicious management, much may be done towards preventing the sufferings of children at this period, by having previously adopted such means as are calculated to develop their physical organization, thereby producing sound constitutions, which would greatly lessen the suffering and danger incident to the process of teething.

When the weather is favorable, young children would be greatly benefitted by being carried out often in the open air; of course exposure should be avoided. But in consequence of a fear of this, children are apt to be kept too closely confined to the house, and are thus debarred the vivifying influence of a pure atmosphere. Those kind mothers, therefore, who feel such a deep solicitude for the welfare of their tender offspring, are not conscious of the injury which they do their children by preventing the very breath of life from fanning their pale and bloodless cheeks. In concluding this portion of our subject, then, we would suggest that a well-regulated diet, cleanliness, and plenty of fresh air, are indispensable requisites to a well ordered nursery.

We will next briefly notice a few of the evils resulting from the system of education as it is generally pursued in our country. It is well known to those who have investigated the matter at all, that long continued mental application at any period of life, will impair the health. But more particularly is this injurious to children, whose nervous systems have not yet acquired sufficient power to support the exhausting influence of protracted study. Yet how frequently do we see children at the ages of six and seven, placed at school under an instructor who is entirely ignorant of the injury which he inflicts upon those under his charge, when he compels them to study eight or ten hours in the day, in the solution of questions in mathematics. What, I would ask, will sooner wear out the constitutional powers of a child at this tender age, than to subject him to such a rigid course of instruction? If the mind is kept in a very active state for a long time, and the brain be too much exhausted by mental labor, all of the other functions will be seriously damaged by a diminished supply of nervous influence; and it is well known that during the period of childhood, all the functions of the body are kept, normally, in a very active condition, especially digestion and nutrition, both of which require for their regular and healthful performance a constant supply of nervous force. When, therefore, we recollect the intimate relation existing between these functions and the rest of the body, in a state of health and disease, it is easy to understand how an over-taxing of the brain may, by cutting off this regular supply of nervous influence to the organs of digestion and assimilation, become the starting point of a number of diseases.

While, therefore, we strongly urge the necessity of early moral and mental training, we should, at the same time, beware of, and condemn any course calculated to make too great a demand upon the mental faculties, which may produce such derangements of the nervous system as may ultimately eventuate in serious consequences.

The health of children at school would be greatly benfitted by keeping them at their tasks for shorter periods, and taking advantage of the intervals for exercise, amusements, &c. By the adoption of measures of this kind, the physical development of children would be greatly increased, which very much facilitates the acquisition of knowledge. In this progressive age, however, the education of children is hurried forward at a rapid rate, even before the mind has had time to expand sufficiently to appreciate its importance. The consequence is, that numbers are turned out annually from our seats of learning, with empty heads and broken constitutions, to take upon themselves the responsibilities of life. These are matters which not only affect health, but affect us nationally, and it is highly important, therefore, that some action should be taken whereby this evil may be corrected, and our intelligence and strength, as a nation, preserved to bless generations yet unborn. Having reached the period of adult life, a new element has now been added to those which affect health in childhood, in the influence of the passions; and it is upon their efficient, or non-efficient, control during youth, that the health and happiness of life, in some degree, depend.

To a certain extent the influence of the passions are beneficial to health. The love of pleasure, hope, ambition, &c., gives buoyancy to life, and imparts vigor to the nervous system. Over-indulgence in ambition, however, is a fruitful source of disease. For the young aspirant, as he launches forth his frail barque upon the stormy sea of life, thinks not of health; but, buoyed up with ambition's delusive charms, he hurries on, denying himself the necessary respite from the cares and perplexities of life. His sleep is unrefreshing, disturbed, perhaps, with wild speculations of the future; and his whole mind is absorbed night and day, in making plans for the consummation of some future object. It may be that he pictures to himself a day of ease and comfort, when he will withdraw from the turmoil of life, and enjoy the profit of his many years labor. But, alas! that future day comes laden with infirmities of the flesh, whose causes can often be traced far back into the past, to some violation of nature's laws, which was disregarded at the time, in the eagerness to gain "the summit of earthly fame, and write thereon a deathless name." The desire of wealth has become the mania of the day, and instead of being regarded as the means of comfort, its true object is often lost sight of, and it becomes in many instances the source of misery. Everything is sacrificed to its attainment, and often when success has crowned the efforts of its votary, a weakened mind and diseased body remain of him, who, having means to obtain every delicacy, finds himself incapable of enjoying the luxuries which now surround him. And thus have thousands sacrificed themselves at ambition's shrine, and thousands more are rushing on with mad-cap zeal to share the same unhappy fate.

But I do not wish to be understood as cultivating, or in any way supporting the doctrine of epicures, the Grecian Philosopher, who believed that the indulgence of the appetite constituted the greatest amount of happiness; on the contrary, I believe that every one should have some object in veiw to accomplish, something to live for, and it is only the overworking of body, and mind, for the accomplishment of this object, that is detrimental to health, and which we condemn as decidedly wrong.

It has been truly said, that the cultivation of the mind, should be the prime object of life; and the fact that man is capable of raising himself far above the rest of the animal kingdom intellectually, would seem to establish the correctness of this belief. Certain it is that a well regulated and enlightened mind adds very much to our earthly happiness, and prepares us for a proper appreciation and preparation for that future state to which we are all fast hastening. But we cannot expect to have a sound mind in an unsound body, both being mutually dependent upon each other. It is important, therefore, that we so act, and so live, as that we may secure for ourselves that happy state, a sound mind in a sound body. This may be acquired by temperance, a well regulated diet, exercise, combined with a contented mind.—Temperance, in its broadest signification, should be the ruling principle in all our actions. The temperate use of ardent spirits should never be lost sight of; for the evils resulting from this species of intemperance are exceedingly injurious to both body and mind, invading the sanctuary of reason and degrading man, the noblest of God's creatures, to a level with the beasts of the field. Nothing can be more injurious to health than an improper use of ardent spirits. The nerves of the strongest men are made to quake and tremble beneath the tread of King Alcohol. And some of the brightest geniuses who have ever adorned the pages of history, have fallen victims to the curse of intemperance. Yet, strange as it may seem, men will persist in the abuse of Alcohol, even at the sacrifice of health and happiness, and not even the wailings of distressed widows, and the cry of orphan children, can stay the hand of the hideous monster, in his ceaseless work of death.

We deem it unnecessary here to enter into a detailed discription of the pathological condition resulting from an intemperate use of alcoholic liquors. Suffice it to say, that it seems to exert its baneful influence principally upon the brain, stomach, and liver—organs, whose regular functions are necessary to a preservation of the body in a state of health, and any interruption of which cannot fail to generate diseased action in some portion of the body. The habit of tobacco-chewing, is also pernicious to health, although it is regarded by two-thirds of our race, as an indispensable luxury. Such is the powerful influence which this agent has over the nervous system, that it is sometimes used medicinally, when there is a necessity for great relaxation of the nervous and muscular systems. It is but rational to infer, therefore, that any subtance capable of exerting such an influence upon the nervous system, must necessarily prove injurious to health when constantly used. Those who have chewed, or smoked this poisonous weed for a length of time, find it almost an impossibility to leave off the habit; notwithstanding they may be convinced that it is injuring them all the while.—This is but another evidence, in proof of the fact, that tobacco does, when used habitually, exert a baneful influence upon the nervous system, and through this medium upon the health of the individual. Another very common source of disease is irregularities in diet. It is important, therefore, that we should begin early in life, to establish certain rules for the regulation of our diet; and in doing so, there are many circumstances to be taken into consideration. For instance, it has been ascertained, that those persons who live in cold climates and lead an active life, require a diet consisting of a large amount of animal matter. The carbon and and hydrogen of which, by being burned off in the lungs, tend to preserve the animal temperature from sinking below a heathly standard. In accordance with this law, we see the Esquimaux subsisting principally upon whale blubber and Seals, two substances which contain a large amount of this heat-generating material. On the other hand, those who live in warm climates do not, for obvious reasons, require such a large quantity of animal matter, and subsist for the most part upon a vegetable diet. Situated then as we are, in a temperate latitude, it would seem that a diet midway between these two extremes, is the one which we should choose. It is well known, that persons who live in warm climates are peculiarly liable to hepatic derangements, which is probably owing to the liver being kept in a state of prolonged excitement by the intense heat and other causes.

It is also a well-established fact, that the liver has for a part of its function the elimination of hydro-carbonaceous matter from the system. The deduction is clear, therefore, that if a large quantity of this non-nitrogenized aliment is taken into the stomach as food, the liver will be inordinately taxed for its elimination; and that this is, in all probability, a frequent cause of hepatic diseases in warm climates. Of course I do not mean to say that this is the only or the principal cause in the production of these diseases. But may not this constant demand made upon the liver for the elimination of this hydro-carbonaceous matter from the system make this organ more liable to become diseased from other causes—such as miasma, heat, sudden transition from heat to cold, &c.?

The quantity of food should be regulated by the age, the amount of exercise, &c. One who is taking active exercise in the open air, and who expends a large amount of muscular and nervous power, requires more food than another who is leading a sedentary life. If, therefore, contrary to the dictates of nature and reason, the denizens of our towns and villages supply their stomachs with as much food as if they were taking active exercise in the open air of the country, the consequence will be indigestion and its concomitant evils.

The preparation or cooking of food is of no little consequence to the health of every one, the great desideratum being to render it, of whatever kind, easy of digestion; and to accomplish this, in a perfect manner, it requires the intervention of some degree of intelligence and art.

The stomach is the great receptacle of food, and the medium through which its nutrient particles gain access to the system for the purpose of building up and repairing the wear and tear which is continually going on in the body; so that a little reflection will show the importance of supplying this organ with good and wholesome food; for if this be unwholesome in its character, or rendered so by the art of cooking, the stomach will be insufficiently supplied with nutrient matter, and emaciation will result, if this state of things is kept up for any length of time.

Exercise is also an important Hygienic agent, and one which is too frequently neglected by many persons, especially females. The most illiterate of the human family are conscious of its beneficial effects—all have tasted the sweets of a keen appetite, and a sound slumber, after a day's exercise. It gives tone and strength to the nervous and muscular systems. Exercise also increases the action of the skin, by which the internal organs are relieved of a portion of their labor; for it is known that when we increase the insensible to sensible perspiration, the secretion from the kidneys is considerably diminished; and this acts beneficially, perhaps, by affording these organs a period of comparative repose. Exercise in the open air, also favors the introduction of oxygen into lungs, which is so essential to the due aeration or oxygenation of the blood; and this is important; for unless the blood is properly submitted to the action of the oxygen of the air during its passage through the lungs, disease will certainly follow sooner, or later. Hence the importance of having our houses so constructed, as that we may always have well ventilated apartments; for if the air in an occupied chamber is not constantly supplied with pure air from the external world, we soon have that within the chamber, exhausted of its oxygen, and surcharged with carbonic acid gas, circumstances which are exceedingly injurious to health. The effects of which are illustrated in the irresistable desire to sleep, which all have experienced in crowded halls, where due attention has not been paid to ventilation; and a neglect of this important hygienic principle is doubtless largely concerned in the production of many diseases among the poor, who from necessity are forced to reside in ill-ventilated houses.

We have thus attempted gentlemen, to present for your consideration a few of the most prominet facts connected with hygiene. There are others which we might refer to, but it is impossible for us to do more than present a brief outline of some of the most important truths connected with this subject. It is one of an interesting nature, and we trust that the day is not far distant, when its beneficial effects may be more fully felt, and realized.

